# FDG-PET as a predictive biomarker for therapy with everolimus in metastatic renal cell cancer

**DOI:** 10.1002/cam4.102

**Published:** 2013-07-10

**Authors:** James L Chen, Daniel E Appelbaum, Masha Kocherginsky, Charles L Cowey, Wendy Kimryn Rathmell, David F McDermott, Walter M Stadler

**Affiliations:** 1 Departments of Biomedical Informatics and Internal Medicine, The Ohio State UniversityColumbus, Ohio; 2 Department of Radiology, The University of ChicagoChicago, Illinois; 3 Department of Health Studies, The University of ChicagoChicago, Illinois; 4 Texas Oncology, PA, Baylor University Medical CenterDallas, Texas; 5 Department of Medicine, University of North Carolina at Chapel HillChapel Hill, North Carolina; 6 Division of Hematology/Oncology, Beth Israel Deaconess Medical CenterBoston, Massachusetts; 7 Department of Medicine, The University of ChicagoChicago, Illinois

**Keywords:** Kidney carcinoma, mTOR protein, pharmacological biomarkers, positron emission tomography

## Abstract

The mTOR (mammalian target of rapamycin) inhibitor, everolimus, affects tumor growth by targeting cellular metabolic proliferation pathways and delays renal cell carcinoma (RCC) progression. Preclinical evidence suggests that baseline elevated tumor glucose metabolism as quantified by FDG-PET ([^18^F] fluorodeoxy-glucose positron emission tomography) may predict antitumor activity. Metastatic RCC (mRCC) patients refractory to vascular endothelial growth factor (VEGF) pathway inhibition were treated with standard dose everolimus. FDG-PET scans were obtained at baseline and 2 weeks; serial computed tomography (CT) scans were obtained at baseline and every 8 weeks. Maximum standardized uptake value (SUVmax) of the most FDG avid lesion, average SUVmax of all measured lesions and their corresponding 2-week relative changes were examined for association with 8-week change in tumor size. A total of 63 patients were enrolled; 50 were evaluable for the primary endpoint of which 48 had both PET scans. Patient characteristics included the following: 36 (72%) clear cell histology and median age 59 (range: 37–80). Median pre- and 2-week treatment average SUVmax were 6.6 (1–17.9) and 4.2 (1–13.9), respectively. Response evaluation criteria in solid tumors (RECIST)-based measurements demonstrated an average change in tumor burden of 0.2% (−32.7% to 35.9%) at 8 weeks. Relative change in average SUVmax was the best predictor of change in tumor burden (all evaluable *P* = 0.01; clear cell subtype *P* = 0.02), with modest correlation. Baseline average SUVmax was correlated with overall survival and progression-free survival (PFS) (*P* = 0.023; 0.020), but not with change in tumor burden. Everolimus therapy decreased SUVs on follow-up PET scans in mRCC patients, but changes were only modestly correlated with changes in tumor size. Thus, clinical use of FDG-PET-based biomarkers is challenged by high variability.

In this phase II trial, FDG-PET was explored as a predictive biomarker for response to everolimus (mTOR inhibition) in metastatic renal cell carcinoma. Everolimus therapy decreased SUVs on follow-up FDG-PET scans in these patients. SUV changes were modestly correlated with changes in tumor size and baseline average SUVmax values were correlated with overall survival.

## Introduction

The oral mTOR (mammalian target of rapamycin) inhibitor, everolimus, affects tumor growth by targeting cellular metabolic proliferation pathways and delays renal cell carcinoma (RCC) progression 1–2. Although everolimus is considered a standard treatment for patients with progression after vascular endothelial growth factor receptor (VEGFR) tyrosine kinase inhibition, identifying patients who have a prolonged response to therapy is critical as the typical response rate is less than 10% and most progress rapidly 1. Therefore, predictive biomarkers are needed to aid clinicians in determining which patients may benefit from mTOR inhibition.

One feature of RCC cells and tumors that has recently garnered significant attention is the altered metabolic properties of these tumors 3. Dysregulated hypoxia signaling 4 and activation of the phosphoinositide 3-kinase pathway 5 make renal carcinoma a natural fit for biomarker studies that incorporate metabolic profiling. These tumors commonly display transcriptional activation of targets of the hypoxia-inducible factor (HIF) such as the glucose transporter, GLUT1 6, rate-limiting glycolytic enzymes 7, and PDK1 8 that blocks pyruvate entry into the Kreb's cycle. Everolimus, an oral mTOR complex-1-targeted agent, is currently an approved single agent for its efficacy to improve progression-free survival (PFS) in patients who have previously been treated with a VEGFR tyrosine kinase inhibitor 2. To this end, murine models suggest that pretherapy elevated tumor glucose uptake as quantified by positron emission tomography (PET) with [^18^F] fluorodeoxy-glucose (FDG-PET) may predict antitumor activity to mTOR inhibition 9 or those patients more likely to achieve a more durable period of disease control.

Moreover, several lines of recently published data have demonstrated a correlation of sequential FDG-PET uptake with the activity of VEGFR tyrosine kinase inhibitors, the other major class of targeted agents used in this disease 10. In one study, time to progression in patients treated with the VEGFR inhibitor, sunitinib 11, was inversely correlated with FDG-PET uptake by the tumors. A second study examined the relationship between FDG-PET uptake and the response in primary tumors to treatment with another VEGFR tyrosine kinase inhibitor, sorafenib, similarly demonstrating that those tumors with the highest avidity for FDG-PET tracer signal were less likely to respond to therapy 12. These studies indicate that the biology of these tumors is likely highly divergent between those that produce a strong metabolic signal with FDG-PET imaging, and those that have more limited glucose uptake. While these relationships may simply reflect a biological indicator of the aggressiveness of the disease, in the setting of examining mTOR inhibitor therapy the potential for altered metabolic programming to directly influence the response to such agents is more mechanistically related.

On the basis of the preclinical data we primarily hypothesized that baseline FDG-PET uptake would correlate with mTOR antitumor activity in renal cancer. We thus conducted a phase II trial to begin to evaluate this hypothesis in the context of oral everolimus therapy – on the basis of the aforementioned clinical data we also planned to explore the relationship between changes in FDG-PET uptake and changes in tumor size and patient outcome. In order to minimize trial size, we chose change in tumor burden at 8 weeks as assessed by response evaluation criteria in solid tumors (RECIST) measurements as the primary endpoint since recent analyses by our group suggested that this metric correlates well with the traditional PFS endpoint.

## Patients and Methods

### Clinical study design

This trial was designed as a single arm, nonblinded phase II study conducted at four sites. Eligible patients were adults with metastatic RCC of any histologic subtype refractory to VEGFR tyrosine kinase inhibitors. Multiple histologic subtypes were allowed because at the time of study initiation, it was known that all histologies apparently benefitted from mTOR inhibition. No prior mTOR inhibitor therapy was allowed, but there were no other restrictions on the number of prior therapies. Patients were required to have a WHO performance status of ≤2, baseline organ function including estimated creatinine clearance of >10 mL/min (by Cockroft-Gault), adequate bone marrow function defined by an absolute neutrophil count ≥1.5 × 10^9^/L, platelets ≥100 × 10^9^/L, hemoglobin >9 g/dL, adequate liver function defined by: serum bilirubin ≤1.5 × upper limit of normal (ULN), and serum transaminases activity ≤3 × ULN (except serum transaminases <5 × ULN if the patient had liver metastases). The trial also required no evidence of viral hepatitis, no evidence of uncontrolled diabetes, and ability to tolerate oral medications. Appropriate informed consent was obtained from patients prior to study initiation.

### Treatment plan

Patients received a fixed dose of everolimus (10 mg) taken orally daily until progression or unacceptable toxicity. Dose reductions to 5 mg daily and 5 mg every other day were specified for grade 3 medication-related toxicity based on National Institutes of Health-National Cancer Institute Common Terminology Criteria for Adverse Events, version 3.0. Grade 4 toxicities resulted in discontinuation of treatment as did a dose delay of greater than 21 days or inability to tolerate 5 mg every other day.

### Imaging

FDG-PET/computed tomography (CT) scans were obtained at baseline (up to 2 weeks prior to start of therapy) and at day 14 (±1) from start of therapy. FDG was obtained commercially and PET protocols were performed with standardized equipment and protocol that account for glucose levels as follows. Patients were fasting for at least 4 h and have a serum glucose concentration of <200 mg/dL. If the serum glucose was **≥**200 mg/dL, delaying the scan 1 day in order to allow medical correction was considered. Insulin was not be used immediately prior to the scan to correct glucose unless medically necessary. In brief, patients were injected with 0.14–0.21 mCi/kg ^18^F-FDG; the dose remaining in the tubing and syringe, as well as any spilled dose, was recorded. Whole body imaging (angle of the jaw to mid-thigh) began 90 ± 10 min after injection using a two- or three-dimensional acquisition mode (as appropriate for the equipment used at each site) with attenuation correction. The PET viewing and analysis software were analyzed using the commercially available PET software MIM (MIM Software Inc., Cleveland, OH). The mean standardized uptake value (SUV) of normal liver, not involved with cancer, was calculated and recorded and used as an internal quality control metric. This value was to verify that changes in measured tumor SUV values were valid across a patient's scans as there are many sources of SUV variability. Serial CT scans were obtained at baseline and every 8 weeks. The treating physician or investigator was blinded to the FDG-PET results.

A single, central reviewer (a nuclear radiologist) evaluated FDG-PET results and was blinded to tumor size evaluations. Maximum SUV (SUVmax) of the most FDG avid lesion, the average SUVmax (avgSUVmax) of all measured lesions, and the corresponding 2-week relative change were examined for association with 8-week change in tumor size. Of note, no a priori criteria for metabolic response or nonresponse were specified and changes in SUV metrics were analyzed as continuous variables.

Patients were reevaluated at their treating site for tumor measurements and response every 8 weeks utilizing multi-detector spiral CT techniques using a 5-mm contiguous reconstruction algorithm of at least the chest, abdomen, and pelvis. All measurable lesions up to a maximum of five lesions per organ and 10 lesions in total, representative of all involved organs, should be identified as target lesions and recorded and measured at baseline. Target lesions were selected on the basis of their size (lesions with the longest diameter) and their suitability for accurate repeated measurements. RECIST 13 measurements were derived from these standard tumor measurements.

### Statistical considerations

The primary endpoint was to explore whether baseline uptake on FDG-PET was associated with changes in tumor burden. Tumor burden was chosen as a primary endpoint as it correlates well with PFS in RCC 14 and requires fewer patient resources 15. Although responses by RECIST criteria are the minority after everolimus therapy, decreases in tumor size are commonly seen 16. High- and low-uptake groups were defined based on avgSUVmax as avgSUVmax >4 and avgSUVmax ≤4 17–18, respectively. The primary endpoint was continuous change in tumor size based on the RECIST criteria from baseline to 8 weeks. Everolimus was expected to be less active in patients with low uptake, and the change in tumor size was expected to be a 20% average increase in the low-uptake group, and a 10% average decrease in the high-uptake group. Based on prior imaging experiences, 70% of patients were expected to have high FDG-PET uptake 17–18. Tumor size changes were expected to be approximately normally distributed when expressed on a logarithmic scale 19–20.

The 20% increase in the low FDG-PET uptake group and the 10% decrease in tumor size in the low- and high-uptake groups correspond to a difference of 0.29 on the log scale. Our data from patients with metastatic renal cell cancer treated with sorafenib 20 suggested that the standard deviation of the mean percent change in tumor size was 33%, which corresponds to SD = 0.40 on the log scale. A total sample size of 60 patients (42 high uptake and 18 low uptake) was planned to provide 81% power to detect a difference of this magnitude with a two-sided α = 0.10 based on a two-sample *t*-test. A planned secondary objective was to correlate the changes in FDG-PET uptake at 2 weeks with changes in tumor size using Pearson correlation coefficient. A sample size of 60 patients would provide 80% power to detect a true correlation of 0.31 or higher, based on a two-sided α = 0.1. Another secondary objective was to determine these associations specifically in patients potentially with von Hippel Lindau pathway inactivation, which was done by separately examining the subset of patients with clear cell carcinoma.

The relationship between uptake parameters and tumor size changes were further analyzed using linear regression. Fisher's exact test was used to test for independence of two categorical variables. PFS and overall survival (OS) were estimated using the method of Kaplan–Meier, and differences between groups were assessed using the log-rank test. Cox proportional hazards regression models were used to examine the effect of uptake parameters as continuous variables, and to estimate hazard ratios (HRs). Average SUVmax across all lesions was used for primary analyses and SUVmax was examined in exploratory analyses.

## Results

### Recruitment

**Figure 1:**
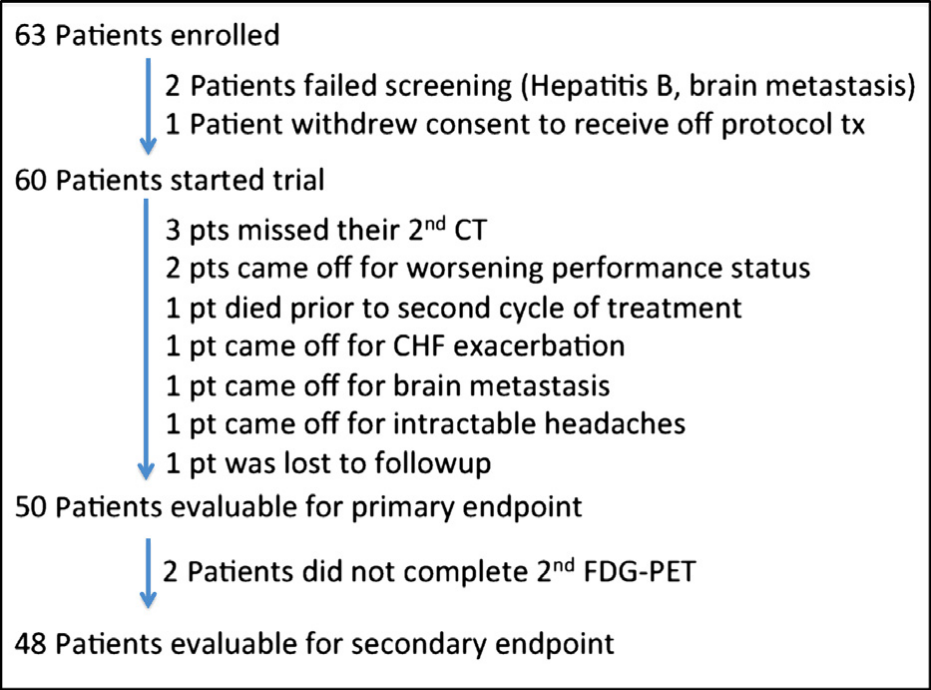
Patient disposition of this clinical trial exploring FDG-PET as a predictive biomarker for therapy with everolimus in metastatic renal cell carcinoma.

Participants were recruited from December 2007 to June 2010. Sixty-three patients signed consent. There were two screen failures and one patient who never began the trial. Thus, as proposed, 60 patients finished baseline screening and started treatment. Fifty patients completed both CT scan time points (enrollment, week 8) and a baseline FDG-PET scan (see Fig. 1) and 48 completed the second FDG-PET scan for secondary endpoint analysis.

### Patient characteristics

Median patient age was 60 (70%) with a male predominance. Of the 50 evaluable patients, the histologic breakdown was as follows: 36 clear cell (median baseline avgSUVmax = 6.4; range 1.8–17.9), four papillary cell (median avgSUVmax = 7.3; range 4.1–14.2), four chromophobe (median avgSUVmax = 2.1; range 1–8.9), and six unclassified tumors (median avgSUVmax = 10.9; range 6.3–13.7). The median number of target lesions at baseline was three (range 1–9). The majority of patients had at least two lines of prior therapy (with at least one VEGFR tyrosine kinase inhibitor containing regimen) and had a clear cell subtype (72%). There was a difference in the proportion of patients with low uptake among renal cell subtypes (*P* = 0.047) (Table 1). Although avgSUVmax was highly variable, patients with chromophobe tumors were more likely to have avgSUVmax ≤4 (75%), although there were only four such patients.

**Table:** Baseline demographics (evaluable patients, *n* = 50)

Variable	Median (range) or total (%)
Age	60 (36–79)
Sex (female)	15 (30)
Number of lines of previous treatment	2 (1–4)
Tumor type	
Clear cell	36 (72)
Papillary cell	4 (8)
Chromophobe	4 (8)
Unclassified/Other	6 (12)
Baseline SUVmax (all)	
All	8.6 (1.0–20.2)
Clear cell	8.0 (1.8–18.4)
Papillary	11.0 (5.7–18.5)
Chromophobe	2.3 (1.0–9.5)
Unclassified/Other	14.5 (7.5–20.2)
SUVmax ≤4 (all): 18 (36%)	
All	9 (18)
Clear cell	6 (17)
Papillary	0 (0)
Chromophobe	3 (75)
Unclassified/Other	0 (0)
Baseline average SUVmax (all)	
All	6.6 (1–17.9)
Clear cell	6.4 (4.1–14.2)
Papillary	7.3 (4.1–14.2)
Chromophobe	2.05 (1–8.9)
Unclassified/Other	10.885 (6.3–13.7)
Average SUVmax ≤4 (all): 20 (40%)	
All	10 (20)
Clear cell	7 (19.4)
Papillary	0 (0)
Chromophobe	3 (75)
Unclassified/Other	0 (0)

### Response to everolimus

**Figure 2:**
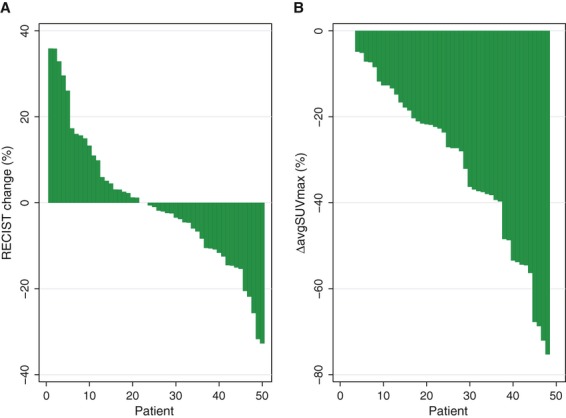
(A) RECIST-based changes in tumor size at 8 weeks post treatment with everolimus are heterogeneous as seen in the waterfall plot. (B) FDG-PET uptake decreased after 2 weeks of treatment for most renal cancer patients.

All patients were treated at the planned dose. The majority of patients (86%) had stable disease at the 8-week time point. The range of responses overall and by tumor subtype is shown in Table 2 and Figure 2A. For an average of 13.6 months (range 1–34.8 months), patients were under follow-up.

**Table:** Response to treatment (RECIST-based tumor size changes at 2 months)

Subtype	Percent change in tumor size (mean, range) and RECIST response category (*n*, %)
All subtypes (*n* = 50)	−0.18% (−32.7% to +35.9%)
Progression of disease	5 (10%)
Stable disease	43 (86%)
Partial response	2 (4%)
Complete response	0 (0%)
Clear cell (*n* = 36)	−2.0% (−32.7% to +35.9%)
Progression of disease	3 (8.3%)
Stable disease	31 (86.1%)
Partial response	2 (5.6%)
Complete response	0
Papillary (*n* = 4)	−1.4% (−14.5% to +16.0%)
Stable disease	4 (100%)
Chromophobe (*n* = 4)	−1.1% (−5.0% to +3.0%)
Stable disease	4 (100%)
Unclassified (*n* = 6)	11.7% (−15.1% to +35.9%)
Progression of disease	2 (33.3%)
Stable disease	4 (66.7%)

The primary analysis explored the association of the log-transformed change in tumor size with the prespecified stratification of high (avgSUVmax >4) versus low (avgSUVmax ≤4) uptake (Table 3). The average change in tumor size in the high- and low-uptake groups was −0.57% (range from −32.7% to 35.9%) and 1.4% (range from −31.7% to 17.3%), respectively (*P* = 0.69; *t*-test on the log scale). As a sensitivity analysis, patients were classified into low- and high-uptake groups based on the median baseline SUVmax among evaluable patients, but no differences in tumor size change were found (*P* = 0.43). We also evaluated avgSUVmax as a continuous predictor, and the relationship was not statistically significant. Similarly, no significant differences were seen for SUVmax.

**Table:** Correlation of FDG-PET uptake measures and tumor size changes (∆log(RECIST))

FDG metric	Statistic/*P*-value (all patients)	Statistic/*P*-value (clear cell only)
Baseline SUVmax (≤4 vs >4)^1^	NS	NS
Baseline SUVmax (continuous)^2^	NS	NS
Baseline avgSUVmax (continuous)^2^	NS	NS
∆%SUVmax (continuous)	NS	NS
∆%avgSUVmax (continuous)	*P* = 0.01	*P* = 0.03

SUVmax, maximum standardized uptake value (SUVmax) of the most FDG avid lesion; aveSUVmax, the average SUVmax of all measured lesions; ∆%SUVmax, percent change from SUVmax at baseline to 2 week reevaluation; ∆%aveSUVmax, change from aveSUVmax at baseline to 2 week reevaluation; NS, not significant.

1 *t*-test.

2 Linear regression, log-transformed measure.

The relationship between early changes in SUV uptake and tumor size changes were examined using linear regression models and log-transformed tumor size changes. Average SUVmax changes (Fig. 2B) ranged from −75.3% to 0% and showed a modest but statistically significant correlation with changes in tumor size (*P* = 0.013; *R*^2^ = 0.13) (Table 3). This effect was seen in the clear cell only cohort (*n* = 35) as well (*P* = 0.027, *R*^2^ = 0.14). Early changes in SUVmax from baseline to 2 weeks ranged from −74.8% to 76.9% (an increase occurred in a single patient), and were not significantly associated with subsequent tumor size change at 8 weeks (*P* = 0.12; *R*^2^ = 0.05).

**Figure 3:**
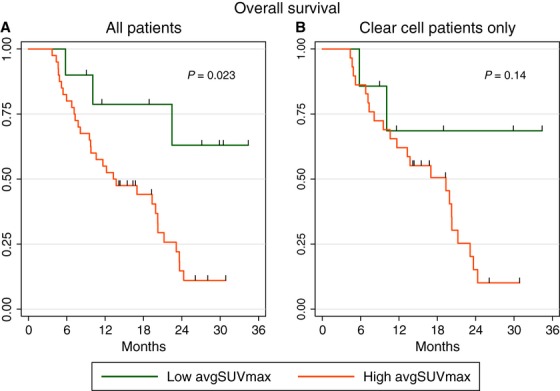
Kaplan–Meier estimates of overall survival among patients with high (>4) and low (≤4) baseline avgSUVmax. (A) Significant difference in overall survival among all patients (*n* = 50). (B) The overall survival difference is no longer statistically significant among the clear cell subtype (*n* = 36).

We explored the association between baseline avgSUVmax and OS and PFS, as well. Patients in the high (avgSUVmax >4) uptake group had worse outcome for both OS (HR = 3.99, *P*=0.023) and PFS (HR = 2.85, *P* = 0.02). The effect remained significant when we compared groups split at the median uptake value both for OS (HR = 2.1; *P* = 0.036) and PFS (HR = 1.91; *P* = 0.046). Although a trend toward significance was noted for OS, this effect disappeared when we examined only the clear cell subtype (HR = 3.0; *P* = 0.14) (Fig. 3).

We then examined the effect of the early relative changes in avgSUVmax. None of the patients had a disease progression or death prior to the second FDG-PET scan, and therefore all patients were included in this analysis. Early changes were not significantly associated with PFS or OS (HR = 1.00 and 0.99, respectively) overall or among the clear cell subtype. Regarding the association of PFS or OS with RECIST-based response, given the low RECIST response rate, there was no correlation between this metric and any FDG uptake metric, either.

## Discussion

This clinical trial evaluated the role of FDG-PET as a biomarker of clinical impact from everolimus therapy in patients with metastatic RCC. Although our initial hypothesis centered on baseline SUVmax as a predictor for change in tumor size, no relationship between these parameters was detectable. Instead, we observed a significant association between baseline SUVmax and OS and PFS. Such a conclusion would need to be confirmed in a larger clear cell cohort. As everolimus does not commonly result in dramatic tumor size changes, future trials may need to consider using a PFS or OS endpoint.

As previously noted, we documented consistent differences in baseline SUVmax among different RCC subtypes. These differences point to marked differences in biology and metabolic programming among renal cell subgroups. Although the numbers of individual tumor types were small for all but the clear cell subgroup, we observed a trend toward increased glucose uptake, consistent with previously suggested increased activation of the mTOR pathway among papillary type RCCs 21. As noted prior by Ho et al. 22, the chromophobe subtype of RCC was noted to have relatively low glucose uptake. Interestingly, patients with higher avgSUVmax had worse outcome for both overall and PFS. This post hoc analysis not only speaks of the heterogeneity among histologic subtypes but also reaffirms that FDG-PET has the potential to be an early noninvasive prognostic measure of outcome 22.

As expected, we did demonstrate a pharmacodynamic effect of everolimus on FDG-PET uptake. However, there was only a modest correlation between changes in glucose uptake and tumor size metrics and no relationship with PFS or OS. This finding recapitulates those published by Kayani et al. They examined clear cell renal cell patients undergoing VEGFR tyrosine kinase inhibition. In their analysis, FDG-PET/CT changes became significantly correlated with RECIST response at 16 weeks follow-up 11 rather than at an earlier 8-week timepoint. Indeed, 86% of patients had stable disease at the first interval imaging point. Therefore, it is possible that if we had repeated a FDG-PET evaluation at a later timepoint, we may have seen a stronger relationship with response to treatment. Nevertheless, this may obviate the clinical predictive value of this expensive biomarker. For example, if we were to use a 16-week endpoint, 13 (26%) of the fifty 8-week evaluable patients in this study who had not come off the trial for drug toxicity would have already reached progression criteria or died before the second FDG-PET evaluation.

Additionally, our clinical findings are also similar to those reported by Ma et al. 23 in which 34 patients with various solid malignancies treated with the related mTOR inhibitor sirolimus experienced a decrease in FDG uptake. As in our trial, however, change in FDG uptake was not predictive of time to progression 24. Taken together, changes in FDG values therefore may not be as relevant as baseline values.

Although we were unable to provide support for the value of FDG-PET as a clinically useful biomarker in the context of mTOR inhibition in renal cancer, a number of limitations should be recognized. The number of fully evaluable patients was less than originally planned and thus the study's power for detecting an effect is modest and extrapolation to survival impact should be performed cautiously. In addition, the study enrolled a heterogeneous group of patients and was not powered to detect the hypothesized effect in the clear cell subgroup. In addition, we were unsuccessful at retrieving six unclassified historical pathologic specimens for further review, and this introduces the potential for inaccuracies in histologic subtyping.

Given these caveats, FDG-PET metrics are only modestly correlated with clinical impact of everolimus on survival endpoints and tumor burden in metastatic RCC, a large effect size is unlikely, and additional studies of this expensive biomarker in renal cancer for this purpose are not recommended. We did observe consistent differences in baseline SUVmax between histologic subtypes, which undoubtedly underlie their unique biological properties. Thus, an important relationship between tumoral FDG avidity and outcome to targeted therapy may exist. Should any future studies of FDG-PET in renal cancer be performed, a histologically uniform group of tumors must be taken into consideration.
